# Effects of Processing and Geometry Parameters on Mass Deviation and Microstructure Evolution in Selective Laser Melted 316L Thin Struts

**DOI:** 10.3390/ma19102011

**Published:** 2026-05-12

**Authors:** Zhongfa Mao, Zhancheng Gu, Yufeng Xie, Wei Guo, Xiulin Ji

**Affiliations:** Key Laboratory of Intelligent Manufacturing Technology of Ministry of Education, College of Engineering, Shantou University, Shantou 515063, China

**Keywords:** 316L stainless steel, fine strut, lattice structure, selective laser melting, microstructure evolution

## Abstract

Selective laser melting (SLM) offers significant potential for fabricating lightweight 316L stainless steel lattice structures (LSs), while forming defects and microstructural heterogeneity remain challenging, especially in fine struts. In this study, response surface methodology (RSM) and analysis of variance (ANOVA) were employed to quantify the coupled effects of geometric parameters (forming angle, FA; rod diameter, RD) and processing parameters (laser power, LP; scanning speed, SS; hatch spacing, HS) on the mass deviation (MD) of fine struts. The results show that FA and RD are the dominant factors affecting MD within the investigated parameter range, whereas LP and SS exhibit comparatively weaker effects. Representative samples with different FA and RD were further characterized by SEM, XRD, and EBSD to examine the associated microstructural evolution. The observations indicate that changes in FA and RD are accompanied by variations in solidification morphology, defect distribution, crystallographic texture, and GND density. Higher FA is associated with lower MD and stronger texture alignment along the building direction, whereas larger RD tends to promote columnar growth and enhanced texture intensity. These results suggest that geometric parameters can serve as effective design variables for tailoring forming deviation and representative microstructural characteristics of fine struts in SLM-fabricated 316L lattice structures.

## 1. Introduction

As the demand for lightweight structures continues to rise across fields like aerospace and automotive engineering, designers are increasingly focused on achieving weight reduction without compromising performance. This is crucial for enhancing fuel efficiency, boosting payload capacity, and optimizing overall operational effectiveness. Lightweighting strategies can be categorized into two main approaches: the use of lightweight materials [1] (e.g., aluminum [2], magnesium [3], titanium alloys [4], and various reinforced composite materials [5]) and the adoption of lightweight structures [6] (e.g., honeycomb [7] and lattice [8] structures). Among these, lightweight structures stand out for their ability to precisely meet specific performance requirements in complex engineering scenarios.

Despite the widespread application of lightweight structures in industrial production, traditional manufacturing methods, such as milling, stamping, and welding, continue to pose challenges. These conventional approaches rely on complex molds and equipment, which increases manufacturing complexity and leads to significant material waste and higher production costs. More critically, they impose severe restrictions on design flexibility, making it challenging to create complex geometric shapes [9,10]. In contrast, additive manufacturing (AM) has emerged as an ideal solution for producing lightweight structures [11]. AM offers numerous advantages, including reduced material waste, enhanced design freedom, shorter production cycles, and the ability to produce high-quality products that meet the stringent performance requirements of industries such as aerospace [12,13,14].

With the continuous advancement of AM technology, there has been a growing interest in lightweight porous materials. Porous structures can be categorized into stochastic (e.g., foams) and ordered (e.g., lattice structures) types [8,15]. Lattice structures (LS), composed of periodically arranged unit cells, have garnered significant attention due to their controllable design and potential applications. By tailoring their topological relationships, lattice structures can be engineered to exhibit superior mechanical properties and specific functional characteristics. These structures enable customized interface interactions, making them ideal for biomedical implants that require osseointegration and aerospace components that demand vibration damping [16,17,18].

Current research on lattice structures primarily focuses on optimizing structural design and processing. Regarding structural design, researchers employ mathematical and CAD techniques to develop optimal topological structures. These strategies have successfully enhanced the strength, stiffness, and energy absorption capabilities of LSs while achieving weight reduction to meet specific engineering requirements [19]. For instance, modifying the shape and connectivity of unit cells can significantly improve the energy absorption capacity of LSs under impact loads. Similarly, optimizing the arrangement of units can enhance compressive strength and vibration-damping performance [20,21]. Additionally, research based on topology optimization theory has provided new insights for LS design, enabling designers to achieve optimal structures under complex loading conditions [22]. For processing optimization, it centers on refining AM processing parameters and post-processing techniques [23,24]. Proper coordination of processing parameters can effectively minimize common structural defects, ensuring the strength and stability of the final product [25,26,27].

However, previous studies have predominantly focused on optimizing either processing parameters or structural parameters individually to improve the formation quality of LSs. The coupled effects of geometric parameters and laser-processing parameters on the forming deviation and representative microstructural characteristics of fine struts remain insufficiently understood. Therefore, this study aims to establish a relationship model between these parameters and mass deviation (MD), and to analyze the associated variations in microstructure, crystallographic texture, and GND distribution in representative samples. The present work is intended to provide experimental evidence for understanding the role of geometric design in the forming behavior of fine struts fabricated by SLM.

## 2. Materials and Methods

### 2.1. Experimental Material and Equipment

**Figure 1:**
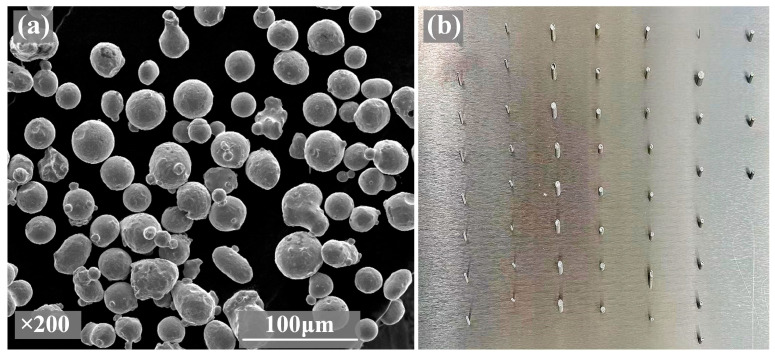
(**a**) The SEM morphology of the raw powder material, and (**b**) corresponding SLMed strut samples.

This study used 316L stainless steel powder supplied by Oerlikon (Oerlikon Technology Co., Ltd., Shanghai, China) as the raw material. The conventional chemical compositions of the powder are presented in Table 1. The powder was reused up to three times; visual inspection and sieving confirmed that the powder remained free of visible oxidation or agglomeration. Figure 1a displays a scanning electron microscope (SEM) image of the powder. Apart from some satellite particles, most of the powder particles are spherical, which contributes to excellent flowability, as indicated by a Hall flowmeter measurement of 4.35 s/50 g. Additionally, the powder exhibits an average particle size of 34.26 μm, a bulk density of 4.27 g/cm^3^, and a vibrationally compacted density of 4.63 g/cm^3^.

The employed selective laser melting (SLM) system was an HBD-280 metal 3D printer (Guangdong Hanbang 3D Tech Co., Ltd., Zhongshan, China). The specific parameters of the SLM system are detailed in Table 2, and the corresponding as-built samples are shown in Figure 1b.

### 2.2. Experimental Procedures

Response Surface Methodology (RSM) was employed to systematically investigate the effects of processing and structural parameters on forming quality and microstructural evolution. RSM is a powerful statistical tool that quantifies the influence of multiple factors on a given response by constructing polynomial models, thereby facilitating the identification of optimal factor combinations for achieving desired outcomes. Consequently, RSM has become a widely recognized and validated optimization method in various mechanical engineering applications. The second-order polynomial response surface model was employed in this work and can be expressed as follows (Equation (1)) [28]:

(1) $$Y=\beta_{0}+\sum_{i=1}^{m}\beta_{i}x_{i}+\sum_{i<j}^{m}\beta_{ij}x_{i}x_{j}+\sum_{i=1}^{m}\beta_{ii}x_{i}^{2}+\varepsilon$$

where *Y* represents the response; $x_{i}$ represents the input factors; $\beta_{i}$, $\beta_{ii}$, and $\beta_{ij}$ are undetermined coefficients; $\beta_{0}$ is the constant term, and ε is the error.

All specimens were fabricated in a nitrogen atmosphere to minimize oxidation (less than 0.1%). The primary processing and structural parameters investigated included laser power (LP), scanning speed (SS), hatch spacing (HS), forming angle (FA), and rod diameter (RD). A standard raster scanning strategy was employed, with a rotation of 67° between consecutive layers to ensure uniform energy distribution. The layer thickness was maintained at 30 μm, and the preheating temperature was fixed at 40 °C to stabilize the building environment.

For RSM analysis, selecting a suitable factor window is important for obtaining a stable response surface. Therefore, preliminary experiments were conducted to identify the parameter ranges. These parameters were designed according to the central composite design (CCD) principle with α = 1.682, enabling comprehensive statistical analysis of the effects of processing and structural parameters on mass deviation (MD) using Minitab (17.1.0) software. A five-factor CCD was constructed, consisting of 32 factorial points, 10 axial points, and 10 centre point replicates, giving a total of 52 experimental runs. The run order was completely randomized to avoid systematic drift. The response variable MD is defined as the relative mass deviation: MD (%) = [(m_fabricated_ − m_CAD_)/m_CAD_] × 100%, where m_fabricated_ and m_CAD_ are the measured and CAD nominal masses, respectively. Experimental error was quantified as the pooled standard deviation from the 10 centre point replicates. The fitted second-order model was validated through analysis of variance (ANOVA), lack-of-fit test (*p* > 0.05), coefficient of determination (R^2^), adjusted R^2^, predicted R^2^, and graphical residual diagnostics (normal probability plot and residuals predicted plot), confirming the assumptions of normality and constant variance. The coded values and corresponding levels of the input factors are detailed in Table 3.

### 2.3. Specimens Characterization

In this study, the actual weight of the fabricated specimens was measured using a BSM-220.4 electronic balance (Zhuojing, Shanghai, China). The diameter and height of the SLMed specimens were measured with at least five measurements taken for each parameter to minimize measurement error, and the average values were used for analysis. MD was calculated from the relative difference between the measured and theoretical masses of each specimen.

Samples were prepared using standard metallographic procedures for microstructural examination. Specifically, the samples were mounted, ground, polished, and then etched. The etching solution consisted of 2 mL of HF, 6 mL of HNO_3_, and 90 mL of ethanol. The sample sections were etched for approximately 45 s to reveal the microstructure. Microstructural analysis was performed using a JSM-6360LA field-emission scanning electron microscope (SEM, JEOL, Tokyo, Japan), which provided high-resolution images that facilitated the observation of grain structure, porosity, and other microstructural features. X-ray diffraction (XRD) measurements were performed on strut samples using a Rigaku Ultima IV diffractometer (Rigaku, Tokyo, Japan) with Cu Kα radiation (λ = 1.5406 Å). The 2θ range was 5–90°, with a step size of 0.02° and a scan speed of 2°/min. Additionally, crystallographic information was obtained using an S-3400N scanning electron microscope (Hitachi, Tokyo, Japan) equipped with an electron backscatter diffraction (EBSD) module. EBSD data were acquired using an Oxford Instruments AZtec (v5.0) system (Oxford Instruments, Abingdon, UK) in regular-grid mode, with an accelerating voltage of 20 kV and a specimen tilt angle of 70°. A constant step size of 1 μm was used for all representative samples. The map dimensions were 1130 × 395 pixels for the samples. Two phases, γ-Fe (fcc) and α-Fe (bcc), were included in the indexing procedure. Grain statistics and pole figure/inverse pole figure maps were generated in MTEX (half-width 10°, cluster size 5°). Geometrically necessary dislocation (GND) maps were calculated from the EBSD orientation gradients using the entrywise norm of the Nye tensor, as implemented in the ATEX (v4.14) software. The calculation parameters were a maximum disorientation of 5.0°, a smoothing factor of 2, and a Burgers circuit size of 3. The normal of the Burgers vector was set parallel to the map normal (z-direction).

## 3. Results and Discussion

### 3.1. Analysis of Variance

Table 4 presents the experimental design matrix and the measured mass deviations (MD) of the SLMed 316L stainless steel specimens. MD was measured as an indicator of forming quality under varying processing and structural parameters. To elucidate the relationship between these parameters and MD, an analysis of Variance (ANOVA) was performed; the results are summarized in Table 5.

In ANOVA, the F-value and *p*-value serve as critical metrics for evaluating the statistical significance of parameter effects on the response variable (MD). The *p*-value quantifies the probability that the observed F-value could occur due to random variation. Factors with a *p*-value less than 0.05 are considered statistically significant, indicating a low probability that their observed effect is due to random variation, meaning there is sufficient evidence to conclude that the factor has a significant effect on the response variable. A *p*-value below 0.01 denotes a highly statistically significant effect. The ANOVA results (Table 5) reveal that hatch spacing (HS), forming angle (FA), and rod diameter (RD) exhibit significant effects on MD (*p* < 0.05). Notably, RD demonstrates a highly significant influence (*p* < 0.01). In contrast, laser power (LP) and scanning speed (SS) only exhibit a minor effect on MD within the studied parameter ranges (*p* > 0.05). The F-values further demonstrate these findings, with RD yielding the highest F-value (80.00), underscoring its dominant influence on MD variation. The adequacy of the developed mathematical model was assessed via the Lack-of-Fit test, which yielded a non-significant *p*-value (0.134 > 0.05). This indicates that the model provides a satisfactory fit to the experimental data and adequately explains the observed MD variations. Based on the significant ANOVA findings, the relationship between the parameters (LP, SS, HS, FA, RD) and MD can be represented by Equation (2). To improve the model’s parsimony and predictive stability, statistically insignificant terms (*p* > 0.5) were removed while maintaining model hierarchy. The refined second-order polynomial model is:

MD = 32.6 − 0.472LP − 0.03297SS + 145.7HS − 0.206FA − 5.31RD + 0.79RD^2^ + 0.000138LP·SS − 0.568LP·HS − 0.0123LP·RD + 0.000073SS·FA − 0.00122SS·RD − 0.735HS·FA + 0.0721FA·RD (2)

The refined model yielded a coefficient of determination R^2^ = 80.51%, an adjusted R^2^ = 73.84%, and a predicted R^2^ = 54.86%. The difference between R^2^ and predicted R^2^ can be attributed to the nature of the response variable MD. In laser powder bed fusion, MD measurements are influenced by several stochastic factors that cannot be controlled by the processing parameters alone, such as partially sintered powder adhesion on surfaces, minor residues from support structures, and local powder spreading inhomogeneities. These effects contribute to an inherent experimental variability that a polynomial model based solely on the main processing parameters cannot fully capture. Despite this, the ANOVA results confirm the high statistical significance of the retained factors (notably RD with an F-value of 80, *p* < 0.01), and the model’s systematic trends provide a reliable basis for identifying key parameters and selecting processing window combinations that minimize MD. The model is therefore considered adequate for factor screening and processing optimization within the investigated range instead of high precision prediction.

### 3.2. Main and Interaction Effects

**Figure 2:**
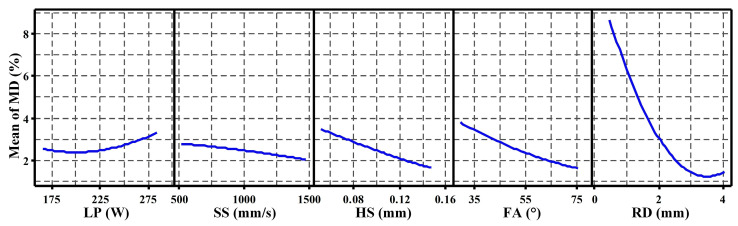
Main effect plots describing the effect of investigated parameters on the sample’s MD.

Based on the RSM analysis, Figure 2 presents the main effects plot, illustrating the influence of individual processing (LP, SS, HS) and structural (FA, RD) parameters on MD. This plot illustrates the mean MD response at various levels of each parameter, while holding the others constant. Consistent with the ANOVA results (Table 5), RD exhibits the most pronounced effect on MD. MD decreases sharply as RD increases up to approximately 3.5 mm. This significant sensitivity at smaller RD may be attributed to the dominant influence of the balling phenomenon on molten pool boundary stability. Beyond 3.5 mm, MD gradually increases with further increases in RD. This trend reversal could stem from factors such as deformation/shrinkage induced by thermal gradients, as well as porosity formation due to molten pool instability, becoming more influential on dimensional accuracy and densification at larger RD.

Moreover, both FA and HS show similar significant effects, with MD decreasing as FA or HS increases. A high FA likely promotes more favorable heat conduction conditions, thereby enhancing melting/solidification stability and surface quality, and ultimately reducing MD. Similarly, a larger HS enables the laser beam to cover a wider area, potentially distributing energy more uniformly and leading to closer agreement between actual and theoretical mass. In contrast, LP and SS demonstrate relatively minor main effects on MD within the studied ranges.

In conclusion, the main-effects analysis identifies RD as the most influential parameter on MD, followed by FA and HS, whereas LP and SS have lesser effects within the investigated parameter space. Lower MD values were associated with RD values around 3.5 mm together with relatively larger FA and HS values. It is worth noting that this trend should be interpreted within the fitted design space rather than as a universally validated optimum. Furthermore, the moderate predicted R^2^ indicates that the model captures systematic trends reliably but is not intended for high precision mass prediction.

**Figure 3:**
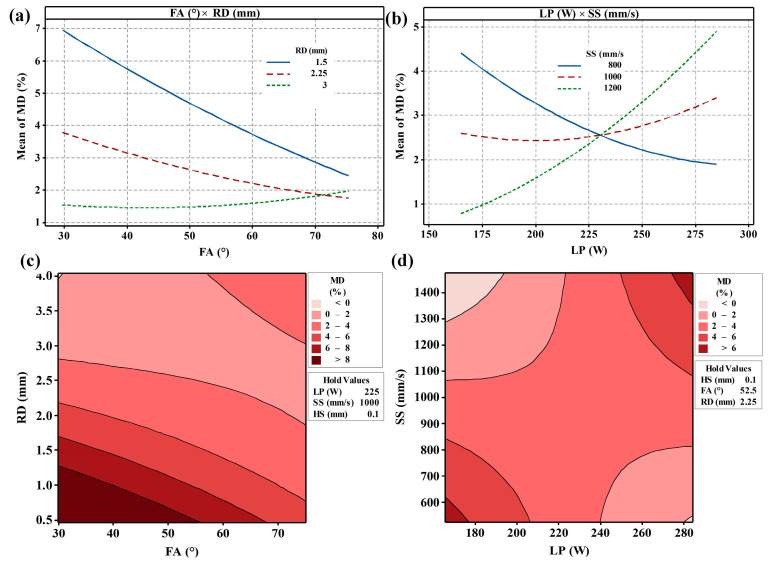
(**a**,**b**) Interaction plots for MD and (**c**,**d**) contour plots of MD from interaction factors FA × RD and LP × SS, respectively. The symbol “×” denotes the interaction term between the corresponding factors in the response surface methodology (RSM) model.

Significant interaction effects between specific parameters were observed, as confirmed by ANOVA and visualized in the interaction plots and contour plots (Figure 3). Notably, strong interactions exist between FA and RD, as well as between LP and SS. For FA and RD (Figure 3a), increasing FA generally reduces MD, likely by improving melt pool stability and heat transfer. Interestingly, increasing RD can effectively mitigate the negative impact of a low FA on MD. Conversely, a high FA diminishes the influence of RD variations, causing MD to converge towards a consistent value. This complex interaction suggests a combined effect of FA and RD on heat transfer pathways and solidification behavior within the struts. For LP and SS (Figure 3b), at a lower SS (800 mm/s), increasing LP adequately melts the powder, reducing MD. However, at higher SS, increasing LP may exacerbate MD. This interaction likely arises from the competing influences of LP and SS on the dynamics of melting and solidification. Higher LP ensures sufficient melting, but higher SS induces a steeper thermal gradient, intensifies Marangoni convection, promotes faster solidification, and increases residual stress, potentially leading to defects and elevated MD. According to the MD contour maps from FA and RD (Figure 3c), the optimized processing window is mainly located in the large RD region and has a relatively large processing range, which provides favorable geometric conditions for reducing MD and improving forming quality. For the MD contour maps from LP and SS (Figure 3d), the optimization region is concentrated in the two opposite intervals of small LP and large SS or large LP and small SS, which provides a direction for the processing optimization of fine strut.

**Figure 4:**
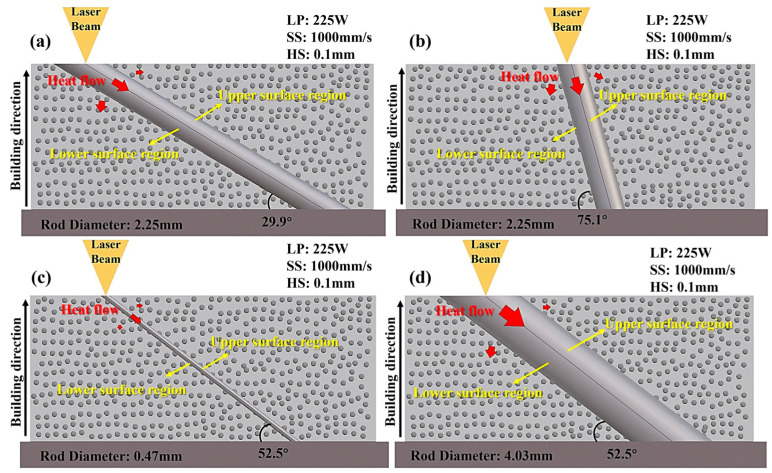
316L stainless steel strut samples’ model images with the numbers (**a**) 39, (**b**) 40, (**c**) 41, and (**d**) 42.

Based on the dominant influence of structural parameters (RD and FA) revealed by the main and interaction effect analyses, these factors were selected for detailed microstructural investigation. Representative samples (Nos. 39, 40, 41, and 42 in Table 4) were chosen because they capture contrasting FA/RD conditions within the design space. Their schematic models are shown in Figure 4. Considering the distinct thermal histories experienced during SLM, each rod sample was examined separately in its upper-surface and lower-surface regions to compare representative microstructural differences associated with local position. It should be noted that the microstructural characterization in the present study was limited to selected representative samples rather than the full DOE matrix.

### 3.3. Phase Identification

**Figure 5:**
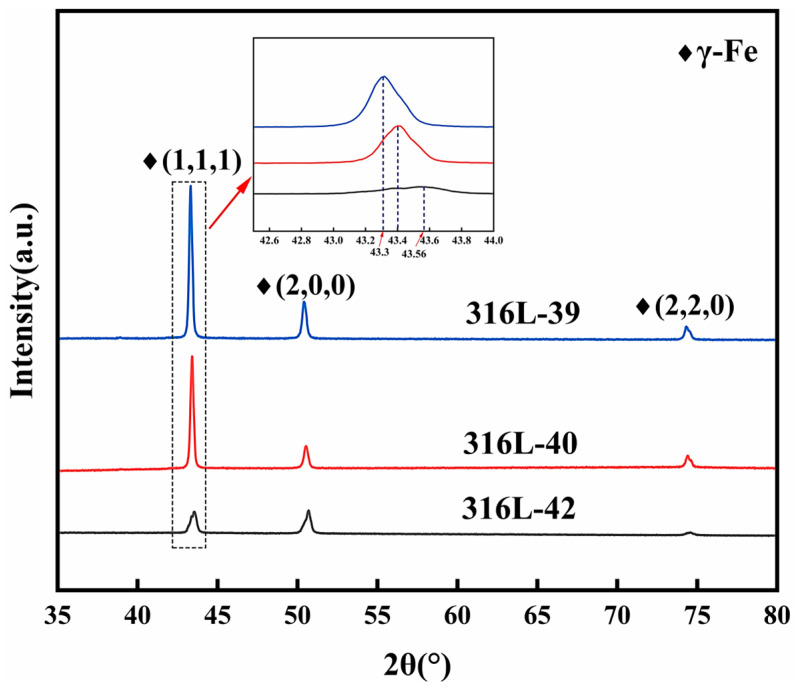
XRD patterns of the SLMed 316L stainless steel strut samples with No. 39, 40, and 42.

X-ray diffraction (XRD) analysis was performed on representative samples (Samples 39, 40, and 42) selected based on their distinct structural parameters (FA and RD; see Table 4 and Figure 4) to investigate the phase composition and potential microstructural variations. Sample 41 was excluded due to insufficient size for a reliable XRD measurement. The obtained XRD spectra are presented in Figure 5.

All analyzed samples exhibit diffraction peaks corresponding to the face-centered cubic γ-austenite phase (FCC, PDF #33-0397), confirming the primary phase constitution of the SLMed 316L stainless steel. However, significant variations in peak intensity and position were observed among these samples, revealing critical microstructural distinctions governed by structural parameters. Specimen 39 (low FA = 29.9°, small RD = 2.25 mm) exhibited substantial (111) peak downshift (43.20°) and intensified texture (I_111_/I_200_) compared to Samples 40 and 42, indicating lattice expansion and formation of a fiber texture (111) parallel to the build direction. In contrast, Specimen 40 (high FA = 75.1 °, small RD = 2.25 mm) exhibits the (111) peak shifting to a high angle, suggesting reduced lattice strain due to the minimized substrate constraint. Moreover, specimen 42 (high FA = 52.5°, large RD = 4.03 mm) only displays slight lattice expansion due to the increase of RD. These results indicate that the FA and RD can modulate lattice structure and texture features through different thermal and constraint conditions during fabrication.

### 3.4. Microstructure Analysis

**Figure 6:**
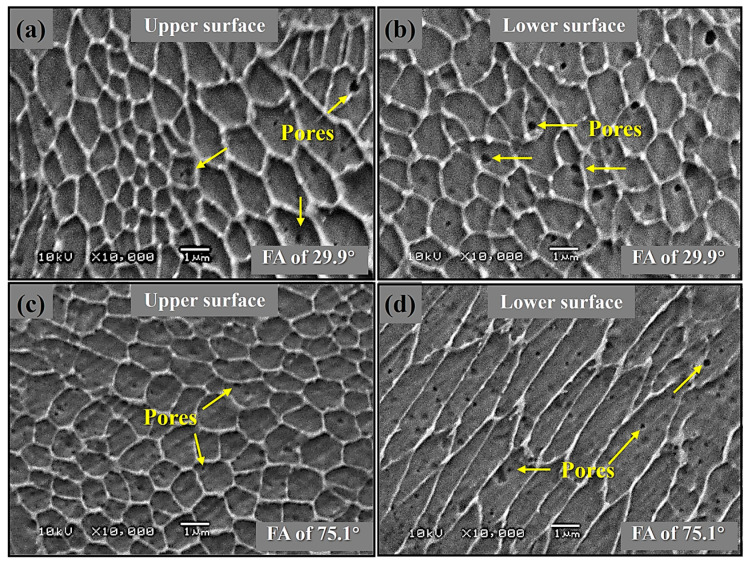
Microstructural SEM images of the upper and lower surface regions for SLMed 316L stainless steel strut samples (**a**,**b**) 39 and (**c**,**d**) 40 with different FA, respectively.

Figure 6 presents comparative SEM microstructures of the upper- and lower-surface regions in representative SLMed 316L specimens with contrasting forming angles (Sample 39: FA = 29.9°; Sample 40: FA = 75.1°). Low-FA strut (Sample 39) exhibits uniform cellular grains but severe vertical defect gradients. The upper surface (Figure 6a) develops fine cellular grains with low porosity due to rapid conductive cooling toward underlying solids. In contrast, the lower surface (Figure 6b) forms coarsened grains with a higher pore density, which is caused by the powder-bed insulation reducing heat conduction and triggering melt pool instability. Notably, the high-FA strut (Sample 40) exhibits a morphological transformation, where cellular grains on the upper surface transition to columnar grains on the lower surface, aligned with the heat flux (Figure 6c,d). This grain evolution originates from the fact that sustained rapid cooling maintains cellular morphology on the upper surface, while steep vertical thermal gradients promote epitaxial growth on the lower surface.

Furthermore, high FA achieves pore reduction on both upper and lower surfaces through favorable axial heat conduction, which stabilizes melt pools and suppresses gas entrapment. As such, this orientation-dependent thermal management directly enables the measured 78% decrease in MD from 5.4% to 1.2% (Table 4) by reducing thermal gradients. This geometry-directed thermal management enables FA to regulate both defect and microstructural morphology.

**Figure 7:**
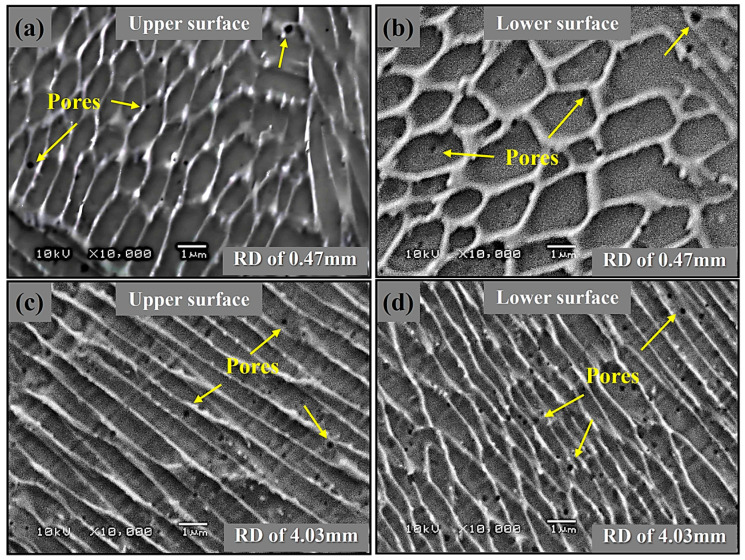
Microstructural SEM images of the upper and lower surface regions for SLMed 316L stainless steel strut samples (**a**,**b**) 41 and (**c**,**d**) 42 with different RD, respectively.

Figure 7 compares solidification microstructures in SLMed 316L struts with identical forming angle (FA = 52.5°) but contrasting rod diameters, i.e., Sample 41 (RD = 0.47 mm) versus Sample 42 (RD = 4.03 mm). It can be found that the rod diameter (RD) can govern a fundamental morphological transition in SLMed 316L lattices. The thin strut (sample 41, Figure 7a,b) undergoes rapid cooling due to the limited heat input in a small cross-sectional area, which stabilizes cellular grains but amplifies powder-interface porosity gradients. In contrast, the thick strut (Sample 42, Figure 7c,d) undergoes bulk-conduction-dominated cooling, allowing for uniform columnar growth along the heat flux direction, but may induce gas entrapment caused by Marangoni convection, resulting in increased porosity. Thus, RD also provides a control strategy for solidification morphology and defect type, allowing lattice-specific designs to meet the needs of various application fields, such as aerospace and biomedical scaffolds.

### 3.5. Crystallographic Texture

**Figure 8:**
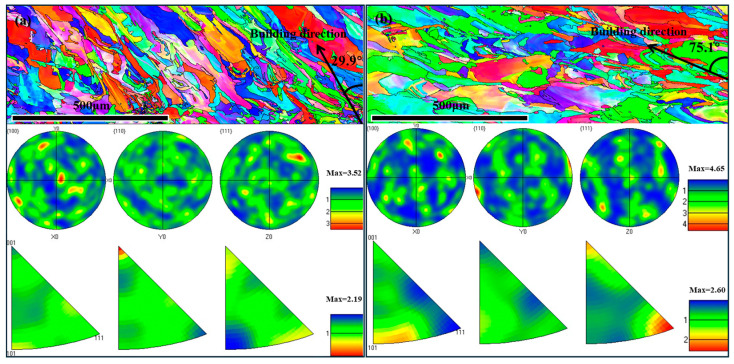
EBSD orientation maps and the corresponding pole figures and inverse pole figures from the top view of samples (**a**) 39 and (**b**) 40, respectively.

Figure 8 presents the electron backscatter diffraction (EBSD) orientation maps and the corresponding pole figures (PF) and inverse pole figures (IPF) for samples 39 and 40, respectively, from the top view. These results provide detailed information on the crystalline texture and grain distribution of the SLMed 316L stainless steel samples. In the orientation maps, different colors correspond to different grain orientations.

EBSD analysis reveals that the forming angle (FA) dominantly controls texture evolution in SLMed 316L lattices. Orientation map (Figure 8a) of low-FA strut (Sample 39) shows color dispersion, meaning that the grains have random orientation and a weak texture, which can also be confirmed by the texture intensity of 3.52 in the PF and 2.19 in the IPF. Interestingly, the grain growth direction has a ~15° deviation from the rod axis direction, indicating that radial heat dissipation suppresses preferential growth. In contrast, the high-FA strut (sample 40) has color dominance areas in the orientation map (Figure 8b), indicating that the texture is enhanced, with texture intensity increasing to 4.65 in the PF and 2.6 in the IPF. Meanwhile, the grains exhibit an epitaxial growth along the building direction with a minimal deviation from the rod axis direction. This texture strengthening originates from FA-governed axial thermal gradients that drive competitive grain selection and eliminate off-axis nuclei. In addition, compared with the low-FA sample, the grain of the high-FA sample becomes coarser due to the intensification of the thermal cycle. Therefore, FA provides a controllable means to tailor the texture strength and crystallographic alignment for the lattice structure.

**Figure 9:**
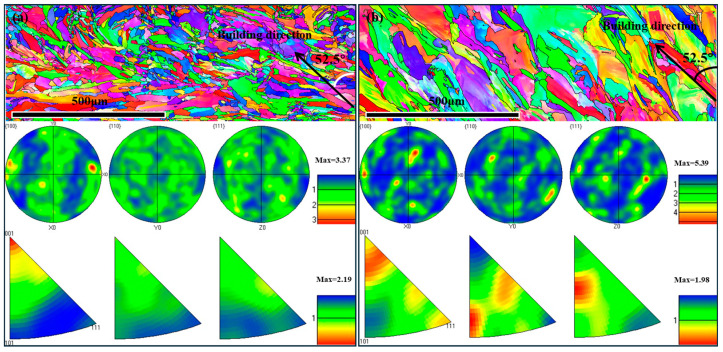
EBSD orientation maps and the corresponding pole figures and inverse pole figures from the top view of samples (**a**) 41 and (**b**) 42, respectively.

Figure 9 shows the EBSD orientation maps and the corresponding pole figure (PF) as well as the inverse pole figure (IPF) of samples 41 and 42. It can be found that the RD dictates texture evolution through thermal regime control. Thin strut (Sample 41, RD = 0.47 mm) exhibits isotropic crystallography with random grain orientations and weak texture intensity (PF: 3.37, IPF: 2.19) in Figure 9a. Crucially, the grains of sample 41 refine noticeably and exhibit a lack of correlation with the building direction, which can be attributed to rapid solidification and multidirectional heat dissipation that suppresses competitive growth. Conversely, the thick strut (sample 42, RD = 4.01 mm) exhibits enhanced texture intensity (5.39), near-ideal alignment with the building direction, and significant grain coarsening, as shown in Figure 9b. This texture enhancement arises from the thick strut’s dominance in heat conduction, enabling sustained axial thermal gradients to drive epitaxial growth, and increased energy input facilitates columnar widening through Ostwald ripening. Thus, RD may be used to tailor the microstructural transition from isotropic in thin struts to strongly textured in thick struts.

### 3.6. Dislocation Density

**Figure 10:**
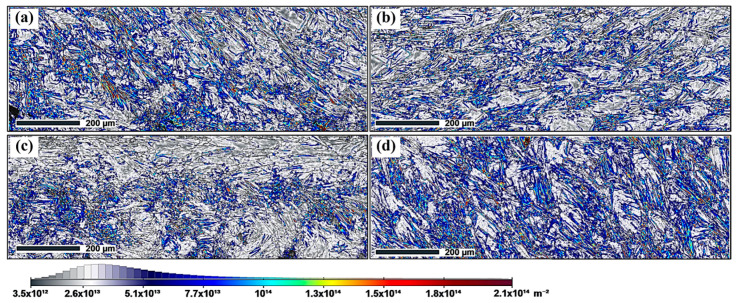
GND maps of different samples (**a**) 39, (**b**) 40, (**c**) 41, and (**d**) 42.

The SLM process, characterized by rapid heating and cooling, significantly influences the dislocation density within the material. The geometrically necessary dislocation (GND) density was calculated for various samples using the ATEX (v.4.14) software. The GND density plots for samples 39, 40, 41, and 42 are presented in Figure 10, and the corresponding average GND densities were calculated to be 5.1 × 10^13^ m^−2^, 4.4 × 10^13^ m^−2^, 4.1 × 10^13^ m^−2^, and 5.7 × 10^13^ m^−2^, respectively. Given that GND values are sensitive to EBSD step size and post-processing parameters, the present results are discussed comparatively among the selected samples rather than as absolute dislocation-density measurements.

Low-FA sample 39 (FA = 29.9°) exhibits a higher GND density than that of high-FA sample 40 (FA = 75.1°). It is worth noting that the dislocations in the low-FA sample are mainly concentrated at the lower surfaces (Figure 10a), which can be attributed to the thermal gradient mismatch between the strut and substrate interfaces and uneven cooling in the molten pool. In contrast, the high-FA sample exhibits a uniform distribution of GND (Figure 10b), as enhanced axial heat flux homogenizes the cooling rates. Thick-strut sample 42 (RD = 4.03 mm) shows 39% higher GND density than sample 41 (RD = 0.47 mm). For a thick strut, the laser energy input in SLM increases due to the increase in forming area in each layer. This prolonged cooling process allows for more time for grain growth, resulting in the formation of additional grain boundaries and dislocations. Moreover, the thick strut also causes a greater accumulation of thermal stresses during cooling, which further promotes the formation of dislocations. Thanks to the thick strut’s dominance in heat conduction, the GND presents a uniform distribution (Figure 10d). In contrast, the thin strut contributes to a central clustering of GND (Figure 10c), which is caused by the central high thermal gradient in the micro-molten pool. Overall, FA and RD emerge as the most influential parameters for microstructural tailoring. The trends in grain morphology and GND density suggest that these two parameters could be used to tune the strength-homogeneity balance, pending confirmation by direct mechanical and residual stress characterization.

## 4. Conclusions

This study systematically investigates the effects of various parameters (laser power, scanning speed, hatch spacing, forming angle, and rod diameter) on the mass deviation of SLMed 316L stainless steel fine strut, and elucidates the evolution law of microstructure under different parameter sets. The key conclusions are the following.

(1) Based on the response surface methodology and ANOVA results, a statistical relationship between the investigated parameters and MD was established. Within the studied parameter range, FA and RD showed significant effects on MD than LP and SS, while HS was also statistically significant.
(2) Microstructural trends suggest that FA may influence thermal gradient distributions and promote microstructural transitions, whereas RD appears to be the primary geometric parameter affecting solidification morphology and dislocation accumulation.
(3) EBSD results indicate that FA and RD are also associated with variations in grain morphology, texture intensity, and crystallographic alignment. Higher FA tended to strengthen alignment along the building direction, while larger RD was associated with increased texture intensity and grain coarsening in the representative samples.
(4) Differences in GND density and distribution were observed among the selected samples, suggesting that geometric parameters may influence local thermal history and deformation heterogeneity during SLM.

Future work, including direct mechanical testing, finite element thermal simulation, quantitative defect statistics, and residual stress characterization, is recommended to validate the hypothesized structure–property relationships and extend the present findings to broader SLM processing windows and other alloy systems.

## Figures and Tables

**Table 1:** The chemical composition of the 316L stainless steel powder.

Elements	Si	Cr	Ni	Mn	Mo	Cu	P	C	O	Fe
Content (wt.%)	0.49	17.60	12.40	1.26	2.61	0.19	0.01	0.02	0.56	Bal.

**Table 2:** Machine specifications and parameters.

Property	Value
Machine	HBD-280
Platform Dimension (L × W × H)	250 mm × 250 mm × 300 mm
Maximum Laser Power	500 W
Layer Thickness	30 μm
Fill Scan Method	Line Scan
Protective Inert Gas	Nitrogen

**Table 3:** Different levels and coded values of processing and structural parameters in the RSM.

Input Factors (Coded Values)	The Levels of Input Factors				
	−1.682	1	0	1	1.682
LP (W)	165.5	200	225	250	284.5
SS (mm/s)	524.3	800	1000	1200	1475.7
HS (mm)	0.052	0.08	0.10	0.12	0.148
FA (°)	29.9	43	52.5	62	75.1
RD (mm)	0.47	1.5	2.25	3	4.03

**Table 4:** Experimental design matrix and measured mass deviations of 316L stainless steel specimens in SLM.

Standard Order	LP (W)	SS (mm/s)	HS (mm)	FA (°)	RD (mm)	MD (%)
1	200	800	0.08	43	1.5	5.3494
2	250	800	0.08	43	1.5	6.0227
3	200	1200	0.08	43	1.5	4.1207
4	250	1200	0.08	43	1.5	5.6879
5	200	800	0.12	43	1.5	5.6828
6	250	800	0.12	43	1.5	3.4392
7	200	1200	0.12	43	1.5	3.5382
8	250	1200	0.12	43	1.5	5.8214
9	200	800	0.08	62	1.5	3.8922
10	250	800	0.08	62	1.5	2.9327
11	200	1200	0.08	62	1.5	2.4831
12	250	1200	0.08	62	1.5	7.28
13	200	800	0.12	62	1.5	4.5592
14	250	800	0.12	62	1.5	1.1357
15	200	1200	0.12	62	1.5	1.3335
16	250	1200	0.12	62	1.5	3.6215
17	200	800	0.08	43	3	2.9217
18	250	800	0.08	43	3	1.6492
19	200	1200	0.08	43	3	0.9203
20	250	1200	0.08	43	3	1.9396
21	200	800	0.12	43	3	2.2736
22	250	800	0.12	43	3	0.4272
23	200	1200	0.12	43	3	0.8938
24	250	1200	0.12	43	3	0.5801
25	200	800	0.08	62	3	2.4209
26	250	800	0.08	62	3	3.1328
27	200	1200	0.08	62	3	2.6059
28	250	1200	0.08	62	3	1.9
29	200	800	0.12	62	3	2.3535
30	250	800	0.12	62	3	0.9314
31	200	1200	0.12	62	3	0.4317
32	250	1200	0.12	62	3	1.8544
33	165.54	1000	0.1	52.5	2.25	2.5018
34	284.46	1000	0.1	52.5	2.25	4.4515
35	225	524.32	0.1	52.5	2.25	3.5112
36	225	1475.68	0.1	52.5	2.25	2.4356
37	225	1000	0.052432	52.5	2.25	3.4398
38	225	1000	0.147568	52.5	2.25	2.7892
39	225	1000	0.1	29.9051	2.25	5.3591
40	225	1000	0.1	75.0949	2.25	1.1506
41	225	1000	0.1	52.5	0.46619	10.612
42	225	1000	0.1	52.5	4.03381	0.5298
43	225	1000	0.1	52.5	2.25	3.3608
44	225	1000	0.1	52.5	2.25	1.969
45	225	1000	0.1	52.5	2.25	3.2512
46	225	1000	0.1	52.5	2.25	1.2256
47	225	1000	0.1	52.5	2.25	3.0148
48	225	1000	0.1	52.5	2.25	3.2632
49	225	1000	0.1	52.5	2.25	1.233
50	225	1000	0.1	52.5	2.25	2.8378
51	225	1000	0.1	52.5	2.25	2.5625
52	225	1000	0.1	52.5	2.25	3.0651

**Table 5:** The results of ANOVA on MDs obtained from different processing parameter sets.

Source	Degree of Freedom	Adjusted Sum of Squares	Adjusted Mean of Squares	F-Value	*p*-Value
Model	20	154.054	7.7027	6.59	0.000
Linear	5	110.990	22.1981	18.99	0.000
LP (W)	1	1.201	1.2011	1.03	0.319
SS (mm/s)	1	1.027	1.0272	0.88	0.356
HS (mm)	1	7.422	7.4218	6.35	0.017
FA (°)	1	7.824	7.8241	6.69	0.015
RD (mm)	1	93.516	93.5161	80.00	0.000
Square	5	12.363	2.4727	2.12	0.090
LP^2^	1	0.415	0.4150	0.35	0.556
SS^2^	1	0.001	0.0013	0.00	0.973
HS^2^	1	0.024	0.0239	0.02	0.887
FA^2^	1	0.118	0.1185	0.10	0.752
RD^2^	1	12.086	12.0859	10.34	0.003
2-Way interaction	10	30.700	3.0700	2.63	0.019
LP × SS	1	15.318	15.3185	13.10	0.001
LP × HS	1	2.580	2.5801	2.21	0.147
LP × FA	1	0.252	0.2524	0.22	0.645
LP × RD	1	1.706	1.7061	1.46	0.236
SS × HS	1	0.056	0.0565	0.05	0.827
SS × FA	1	0.609	0.6093	0.52	0.476
SS × RD	1	1.072	1.0719	0.92	0.346
HS × FA	1	0.625	0.6249	0.53	0.470
HS × RD	1	0.025	0.0249	0.02	0.885
FA × RD	1	8.456	8.4558	7.23	0.011
Error	31	36.236	1.1689		
Lack of Fit	22	30.196	1.3725	2.05	0.134
Pure Error	9	6.040	0.6711		
Total	51	190.290			

The symbol “×” denotes the interaction term between the corresponding factors in the response surface methodology (RSM) model.

## Data Availability

The original contributions presented in this study are included in the article. Further inquiries can be directed to the corresponding authors.
